# Ethyl 1-(2-hy­droxy­eth­yl)-2-(4-meth­oxy­phen­yl)-1*H*-benzimidazole-5-carboxyl­ate monohydrate

**DOI:** 10.1107/S1600536812001262

**Published:** 2012-01-18

**Authors:** Natarajan Arumugam, Nurziana Ngah, Hasnah Osman, Aisyah Saad Abdul Rahim

**Affiliations:** a School of Pharmaceutical Sciences, Universiti Sains Malaysia, 11800 USM, Penang, Malaysia; b Kulliyyah of Science, International Islamic University Malaysia, Kuantan Campus, Jalan Istana, Bandar Indera Mahkota, 25200 Kuantan, Pahang, Malaysia; c School of Chemical Sciences, Universiti Sains Malaysia, 11800 USM, Penang, Malaysia

## Abstract

In the title mol­ecule, C_19_H_20_N_2_O_4_·H_2_O, the benzimidazole ring system is essentially planar [maximum deviation = 0.013 (11) Å] and is inclined to the 4-meth­oxy­phenyl ring by 30.98 (5)°. In the crystal, O—H⋯O and O—H⋯N hydrogen bonds involving the water mol­ecule link neighbouring mol­ecules, forming a two-dimensional network lying parallel to the *bc* plane. There are also C—H⋯π and π–π inter­actions present. The latter involve inversion-related benzimidazole rings with centroid–centroid distances of 3.5552 (8) and 3.7466 (8) Å.

## Related literature

For the synthesis of the title compound, see: Arumugam *et al.* (2010 ▶). For the biological activity of benzimidazole derivatives, see: Cosar & Julou (1959 ▶); Gudmundsson *et al.* (1999 ▶); De Clercq *et al.* (1993 ▶); Spasov *et al.* (1999 ▶). For standard bond lengths, see: Allen *et al.* (1987 ▶).
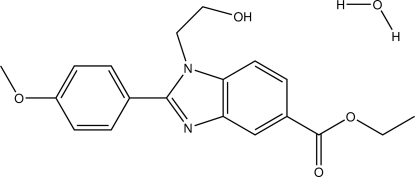


## Experimental

### 

#### Crystal data

C_19_H_20_N_2_O_4_·H_2_O
*M* *_r_* = 358.39
Monoclinic, 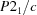

*a* = 10.6364 (11) Å
*b* = 9.5089 (10) Å
*c* = 19.3765 (17) Å
β = 112.899 (5)°
*V* = 1805.3 (3) Å^3^
*Z* = 4
Mo *K*α radiation
μ = 0.10 mm^−1^
*T* = 293 K
0.35 × 0.25 × 0.18 mm

#### Data collection

Bruker SMART APEXII CCD area-detector diffractometer
Absorption correction: multi-scan (*SADABS*; Bruker, 2009 ▶) *T*_min_ = 0.967, *T*_max_ = 0.983
13982 measured reflections
3140 independent reflections
2856 reflections with *I* > 2σ(*I*)
*R*_int_ = 0.033

#### Refinement

*R*[*F* ^2^ > 2σ(*F* ^2^)] = 0.032
*wR*(*F* ^2^) = 0.086
*S* = 1.02
3140 reflections
249 parameters
3 restraints
H atoms treated by a mixture of independent and constrained refinement
Δρ_max_ = 0.19 e Å^−3^
Δρ_min_ = −0.20 e Å^−3^

### 

Data collection: *APEX2* (Bruker, 2009 ▶); cell refinement: *SAINT* (Bruker, 2009 ▶); data reduction: *SAINT*; program(s) used to solve structure: *SHELXS97* (Sheldrick, 2008 ▶); program(s) used to refine structure: *SHELXL97* (Sheldrick, 2008 ▶); molecular graphics: *SHELXTL* (Sheldrick, 2008 ▶); software used to prepare material for publication: *SHELXTL* and *PLATON* (Spek, 2009 ▶).

## Supplementary Material

Crystal structure: contains datablock(s) global, I. DOI: 10.1107/S1600536812001262/su2368sup1.cif

Structure factors: contains datablock(s) I. DOI: 10.1107/S1600536812001262/su2368Isup2.hkl

Supplementary material file. DOI: 10.1107/S1600536812001262/su2368Isup3.cml

Additional supplementary materials:  crystallographic information; 3D view; checkCIF report

## Figures and Tables

**Table 1:** Hydrogen-bond geometry (Å, °) *Cg*2 is the centroid of the C1–C6 ring.

*D*—H⋯*A*	*D*—H	H⋯*A*	*D*⋯*A*	*D*—H⋯*A*
O5—H5*B*⋯O4^i^	0.86 (2)	1.98 (2)	2.8165 (15)	165 (2)
O5—H5*C*⋯N2^ii^	0.85 (1)	1.95 (1)	2.8011 (15)	175 (2)
C15—H15*A*⋯*Cg*2^iii^	0.97	2.95	3.7247 (17)	138

Symmetry codes: (i) 
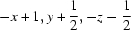
; (ii) 
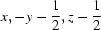
; (iii) 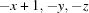.

**Fig. 1.**
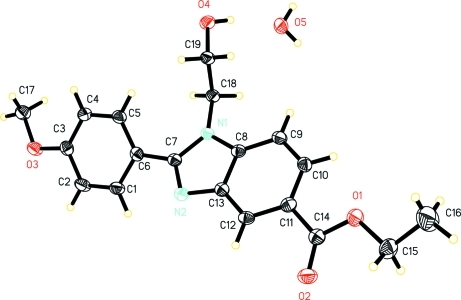
The molecular structure of the title compound, with the atom numbering and displacement ellipsods drawn at the 40% probability level.

**Fig. 2.**
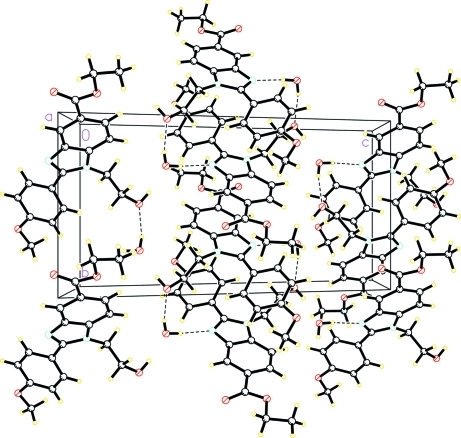
The crystal packing of the title compound viewed along the a axis. The O—H···O and O—H···N hydrogen bonds are shown as dashed lines.

## supplementary crystallographic information

### Comment

Given the significance of benzimidazole as apart of the purine nucleoside framework, drug design based on benzimidazoles is an interesting topic for synthetic medicinal chemists (Spasov *et al.*, 1999). The unique biological activity of *N*-alkylated benzimidazole on treating diseases such as protozoal infections, like trichomoniasis (Cosar & Julou, 1959) as well as nucleoside analogues inhibiting viral infections, like 2,5,6-trichloro-1-(β-*D*-ribofuranosyl)benzimidazole (Gudmundsson *et al.*, 1999) and ribavin (De Clercq *et al.*, 1993), have been reported. Thus, in view of their importance, the crystal structure analysis of the title benzimidazole compound was carried out and the results are presented herein.

The title compound, (Fig. 1), is a benzimidazole derivative and is similiar to the *p*-tolyl derivative, ethyl 1-(2-hydroxylethyl)-2-*p*-tolyl-1*H*-benzimidazole-5-carboxylate, reported on by (Arumugam *et al.*, 2010). The title compound is associated with one water molecule of crystallization. The bond lengths (Allen *et al.*, 1987) and angles are in normal ranges and are comparable to those reported for the *p*-tolyl derivative mentioned above. The benzimidazole ring (N1/N2/C7—C13) is essentially planar with a maximum deviation of 0.013 (11) Å for atom C12. The phenyl ring is inclined at an angle of 30.98 (5)° to the benzimidazole mean plane.

In the crystal, the water molecule links the organic molecules *via* intermolecular O5—H5B···O4 and O5—H5C···N2 hydrogen bonds (Table 1), so forming a two dimensional network lieing parallel to the *bc* plane. An intermolecular C—H···Cg2 interaction is also observed (Table 1), and there are also π–π stacking interactions involving inversion related benzimidazole rings: *Cg*1···*Cg*3^i^ = 3.552 (8) Å, *Cg*3···*Cg*3^i^ = 3.7466 (8) Å [*Cg*1 and *Cg*3 are the centroids of rings (N1/N2/C7/C8/C13) and (C8—C13), respectively; symmetry code: (i) -x+1, -y, -z].

### Experimental

The title compound was prepared according to the method described by Arumugam *et al.* (2010). Colourless block-like crystals of the title compound, suitable for X-ray diffraction analysis, were obtained by slow evaporation of a solution of the title compound in EtOAc

### Refinement

The water and the hydroxy H-atoms were located from difference Fourier map and were freely refined. The C-bound H atoms were included in calculated positions and refined using a riding model: C—H = 0.93, 0.96 and 0.97 Å, for CH, CH_3_ and CH_2_ H-atoms, respectively, with *U*_iso_(H) = k *x U*_eq_(C), where k = 1.5 for CH_3_ H-atoms and k = 1.2 for all other H atoms. A rotating group model was applied to the methyl groups.

### Crystal data

**Table:**

C_19_H_20_N_2_O_4_·H_2_O	*F*(000) = 760
*M**_r_* = 358.39	*D*_x_ = 1.319 Mg m^−^^3^
Monoclinic, *P*2_1_/*c*	Mo *K*α radiation, λ = 0.71073 Å
Hall symbol: -P 2ybc	Cell parameters from 9429 reflections
*a* = 10.6364 (11) Å	θ = 2.9–25.0°
*b* = 9.5089 (10) Å	µ = 0.10 mm^−^^1^
*c* = 19.3765 (17) Å	*T* = 293 K
β = 112.899 (5)°	Block, colourless
*V* = 1805.3 (3) Å^3^	0.35 × 0.25 × 0.18 mm
*Z* = 4	

### Data collection

**Table:**

Bruker SMART APEXII CCD area-detector diffractometer	3140 independent reflections
Radiation source: fine-focus sealed tube	2856 reflections with *I* > 2σ(*I*)
graphite	*R*_int_ = 0.033
Detector resolution: 83.66 pixels mm^-1^	θ_max_ = 25.0°, θ_min_ = 2.9°
φ and ω scan	*h* = −12→12
Absorption correction: multi-scan (*SADABS*; Bruker, 2009)	*k* = −11→11
*T*_min_ = 0.967, *T*_max_ = 0.983	*l* = −23→21
13982 measured reflections	

### Refinement

**Table:**

Refinement on *F*^2^	Primary atom site location: structure-invariant direct methods
Least-squares matrix: full	Secondary atom site location: difference Fourier map
*R*[*F*^2^ > 2σ(*F*^2^)] = 0.032	Hydrogen site location: inferred from neighbouring sites
*wR*(*F*^2^) = 0.086	H atoms treated by a mixture of independent and constrained refinement
*S* = 1.02	*w* = 1/[σ^2^(*F*_o_^2^) + (0.0382*P*)^2^ + 0.7017*P*] where *P* = (*F*_o_^2^ + 2*F*_c_^2^)/3
3140 reflections	(Δ/σ)_max_ < 0.001
249 parameters	Δρ_max_ = 0.19 e Å^−^^3^
3 restraints	Δρ_min_ = −0.20 e Å^−^^3^

### Special details

**Table:**

Geometry. All e.s.d.'s (except the e.s.d. in the dihedral angle between two l.s. planes) are estimated using the full covariance matrix. The cell e.s.d.'s are taken into account individually in the estimation of e.s.d.'s in distances, angles and torsion angles; correlations between e.s.d.'s in cell parameters are only used when they are defined by crystal symmetry. An approximate (isotropic) treatment of cell e.s.d.'s is used for estimating e.s.d.'s involving l.s. planes.
Refinement. Refinement of *F*^2^ against ALL reflections. The weighted *R*-factor *wR* and goodness of fit *S* are based on *F*^2^, conventional *R*-factors *R* are based on *F*, with *F* set to zero for negative *F*^2^. The threshold expression of *F*^2^ > σ(*F*^2^) is used only for calculating *R*-factors(gt) *etc*. and is not relevant to the choice of reflections for refinement. *R*-factors based on *F*^2^ are statistically about twice as large as those based on *F*, and *R*- factors based on ALL data will be even larger.

### Fractional atomic coordinates and isotropic or equivalent isotropic displacement parameters (Å^2^)

**Table:**

	*x*	*y*	*z*	*U*_iso_*/*U*_eq_	
O1	0.03607 (9)	0.11092 (10)	−0.10495 (5)	0.0273 (2)	
O2	0.13828 (10)	0.11955 (10)	0.02067 (5)	0.0318 (2)	
O3	1.05219 (9)	−0.64334 (10)	0.19548 (5)	0.0299 (2)	
O4	0.56061 (11)	−0.49083 (10)	−0.20486 (5)	0.0308 (2)	
H4B	0.5547 (19)	−0.4247 (16)	−0.2358 (9)	0.055 (6)*	
N1	0.54707 (10)	−0.27695 (10)	−0.04766 (5)	0.0195 (2)	
N2	0.53184 (10)	−0.24427 (10)	0.06353 (6)	0.0207 (2)	
C1	0.79606 (13)	−0.37042 (14)	0.14242 (7)	0.0246 (3)	
H1A	0.7743	−0.2930	0.1650	0.029*	
C2	0.90530 (13)	−0.45375 (15)	0.18365 (7)	0.0277 (3)	
H2A	0.9560	−0.4328	0.2337	0.033*	
C3	0.94031 (12)	−0.56962 (14)	0.15058 (7)	0.0235 (3)	
C4	0.86192 (13)	−0.60221 (13)	0.07631 (7)	0.0243 (3)	
H4A	0.8838	−0.6799	0.0540	0.029*	
C5	0.75072 (13)	−0.51868 (13)	0.03529 (7)	0.0231 (3)	
H5A	0.6979	−0.5421	−0.0142	0.028*	
C6	0.71694 (12)	−0.40026 (13)	0.06695 (7)	0.0206 (3)	
C7	0.59923 (12)	−0.30890 (12)	0.02771 (7)	0.0194 (3)	
C8	0.43855 (12)	−0.18642 (12)	−0.06055 (7)	0.0197 (3)	
C9	0.34925 (13)	−0.12055 (13)	−0.12536 (7)	0.0219 (3)	
H9A	0.3561	−0.1332	−0.1714	0.026*	
C10	0.24989 (12)	−0.03538 (13)	−0.11789 (7)	0.0224 (3)	
H10A	0.1875	0.0093	−0.1600	0.027*	
C11	0.24092 (12)	−0.01465 (12)	−0.04771 (7)	0.0217 (3)	
C12	0.33239 (12)	−0.07870 (12)	0.01676 (7)	0.0212 (3)	
H12A	0.3277	−0.0634	0.0631	0.025*	
C13	0.43118 (12)	−0.16652 (12)	0.00957 (7)	0.0196 (3)	
C14	0.13541 (13)	0.07845 (13)	−0.03903 (7)	0.0234 (3)	
C15	−0.06792 (14)	0.20632 (16)	−0.10130 (8)	0.0328 (3)	
H15A	−0.0296	0.2992	−0.0858	0.039*	
H15B	−0.1042	0.1723	−0.0656	0.039*	
C16	−0.17773 (17)	0.2119 (2)	−0.17790 (10)	0.0550 (5)	
H16A	−0.2463	0.2782	−0.1785	0.082*	
H16B	−0.2182	0.1205	−0.1914	0.082*	
H16C	−0.1395	0.2407	−0.2131	0.082*	
C17	1.10325 (14)	−0.74776 (15)	0.15978 (7)	0.0292 (3)	
H17A	1.1895	−0.7819	0.1946	0.044*	
H17B	1.1148	−0.7070	0.1173	0.044*	
H17C	1.0396	−0.8243	0.1435	0.044*	
C18	0.59596 (12)	−0.31970 (13)	−0.10568 (7)	0.0213 (3)	
H18A	0.6892	−0.3533	−0.0822	0.026*	
H18B	0.5952	−0.2393	−0.1366	0.026*	
C19	0.50589 (13)	−0.43538 (13)	−0.15438 (7)	0.0235 (3)	
H19A	0.4971	−0.5104	−0.1226	0.028*	
H19B	0.4155	−0.3979	−0.1826	0.028*	
O5	0.54527 (11)	−0.26408 (11)	−0.28926 (5)	0.0342 (2)	
H5B	0.510 (2)	−0.1856 (14)	−0.2842 (12)	0.062 (6)*	
H5C	0.542 (2)	−0.267 (2)	−0.3338 (7)	0.061 (6)*	

### Atomic displacement parameters (Å^2^)

**Table:**

	*U*^11^	*U*^22^	*U*^33^	*U*^12^	*U*^13^	*U*^23^
O1	0.0236 (5)	0.0305 (5)	0.0275 (5)	0.0048 (4)	0.0096 (4)	0.0009 (4)
O2	0.0326 (5)	0.0363 (5)	0.0274 (5)	0.0044 (4)	0.0126 (4)	−0.0064 (4)
O3	0.0288 (5)	0.0376 (5)	0.0214 (5)	0.0106 (4)	0.0077 (4)	−0.0008 (4)
O4	0.0533 (6)	0.0220 (5)	0.0257 (5)	0.0010 (4)	0.0248 (5)	−0.0003 (4)
N1	0.0216 (5)	0.0199 (5)	0.0179 (5)	−0.0012 (4)	0.0086 (4)	−0.0012 (4)
N2	0.0217 (5)	0.0214 (5)	0.0193 (5)	−0.0026 (4)	0.0085 (4)	−0.0012 (4)
C1	0.0262 (6)	0.0284 (7)	0.0211 (6)	0.0019 (5)	0.0113 (5)	−0.0031 (5)
C2	0.0274 (7)	0.0361 (7)	0.0182 (6)	0.0024 (6)	0.0074 (5)	−0.0032 (5)
C3	0.0216 (6)	0.0276 (6)	0.0220 (6)	0.0015 (5)	0.0092 (5)	0.0030 (5)
C4	0.0282 (7)	0.0207 (6)	0.0246 (6)	−0.0003 (5)	0.0111 (5)	−0.0021 (5)
C5	0.0255 (6)	0.0226 (6)	0.0191 (6)	−0.0044 (5)	0.0065 (5)	−0.0017 (5)
C6	0.0212 (6)	0.0215 (6)	0.0207 (6)	−0.0039 (5)	0.0099 (5)	0.0011 (5)
C7	0.0213 (6)	0.0186 (6)	0.0187 (6)	−0.0057 (5)	0.0083 (5)	−0.0018 (5)
C8	0.0210 (6)	0.0175 (6)	0.0212 (6)	−0.0044 (5)	0.0089 (5)	−0.0026 (5)
C9	0.0262 (6)	0.0221 (6)	0.0182 (6)	−0.0035 (5)	0.0095 (5)	−0.0023 (5)
C10	0.0229 (6)	0.0211 (6)	0.0205 (6)	−0.0024 (5)	0.0055 (5)	0.0001 (5)
C11	0.0211 (6)	0.0188 (6)	0.0246 (6)	−0.0041 (5)	0.0084 (5)	−0.0030 (5)
C12	0.0236 (6)	0.0213 (6)	0.0207 (6)	−0.0062 (5)	0.0109 (5)	−0.0043 (5)
C13	0.0203 (6)	0.0187 (6)	0.0194 (6)	−0.0050 (5)	0.0071 (5)	−0.0015 (5)
C14	0.0232 (6)	0.0214 (6)	0.0252 (7)	−0.0043 (5)	0.0091 (5)	−0.0016 (5)
C15	0.0277 (7)	0.0369 (8)	0.0368 (8)	0.0092 (6)	0.0159 (6)	0.0039 (6)
C16	0.0359 (9)	0.0788 (13)	0.0442 (10)	0.0216 (9)	0.0090 (8)	0.0018 (9)
C17	0.0282 (7)	0.0342 (7)	0.0264 (7)	0.0070 (6)	0.0120 (6)	−0.0001 (6)
C18	0.0252 (6)	0.0225 (6)	0.0195 (6)	0.0001 (5)	0.0123 (5)	0.0004 (5)
C19	0.0302 (7)	0.0231 (6)	0.0189 (6)	−0.0008 (5)	0.0115 (5)	−0.0008 (5)
O5	0.0533 (6)	0.0309 (5)	0.0247 (5)	0.0084 (5)	0.0219 (5)	0.0080 (4)

### Geometric parameters (Å, °)

**Table:**

O1—C14	1.3391 (16)	C9—C10	1.3831 (18)
O1—C15	1.4533 (15)	C9—H9A	0.9300
O2—C14	1.2100 (16)	C10—C11	1.4135 (18)
O3—C3	1.3633 (15)	C10—H10A	0.9300
O3—C17	1.4324 (16)	C11—C12	1.3900 (18)
O4—C19	1.4200 (15)	C11—C14	1.4895 (17)
O4—H4B	0.854 (9)	C12—C13	1.3909 (17)
N1—C7	1.3794 (15)	C12—H12A	0.9300
N1—C8	1.3825 (16)	C15—C16	1.490 (2)
N1—C18	1.4677 (15)	C15—H15A	0.9700
N2—C7	1.3268 (16)	C15—H15B	0.9700
N2—C13	1.3838 (16)	C16—H16A	0.9600
C1—C2	1.3761 (19)	C16—H16B	0.9600
C1—C6	1.4033 (18)	C16—H16C	0.9600
C1—H1A	0.9300	C17—H17A	0.9600
C2—C3	1.3961 (18)	C17—H17B	0.9600
C2—H2A	0.9300	C17—H17C	0.9600
C3—C4	1.3887 (18)	C18—C19	1.5215 (17)
C4—C5	1.3893 (18)	C18—H18A	0.9700
C4—H4A	0.9300	C18—H18B	0.9700
C5—C6	1.3946 (17)	C19—H19A	0.9700
C5—H5A	0.9300	C19—H19B	0.9700
C6—C7	1.4695 (17)	O5—H5B	0.855 (9)
C8—C9	1.3940 (17)	O5—H5C	0.852 (9)
C8—C13	1.4040 (17)		
			
C14—O1—C15	115.49 (10)	C11—C12—C13	117.71 (11)
C3—O3—C17	116.68 (10)	C11—C12—H12A	121.1
C19—O4—H4B	105.8 (13)	C13—C12—H12A	121.1
C7—N1—C8	106.98 (10)	N2—C13—C12	129.62 (11)
C7—N1—C18	129.31 (10)	N2—C13—C8	109.94 (10)
C8—N1—C18	123.63 (10)	C12—C13—C8	120.44 (11)
C7—N2—C13	105.60 (10)	O2—C14—O1	123.68 (12)
C2—C1—C6	121.14 (12)	O2—C14—C11	124.06 (12)
C2—C1—H1A	119.4	O1—C14—C11	112.26 (10)
C6—C1—H1A	119.4	O1—C15—C16	106.87 (12)
C1—C2—C3	120.24 (12)	O1—C15—H15A	110.3
C1—C2—H2A	119.9	C16—C15—H15A	110.3
C3—C2—H2A	119.9	O1—C15—H15B	110.3
O3—C3—C4	124.72 (11)	C16—C15—H15B	110.3
O3—C3—C2	115.81 (11)	H15A—C15—H15B	108.6
C4—C3—C2	119.47 (11)	C15—C16—H16A	109.5
C3—C4—C5	119.97 (12)	C15—C16—H16B	109.5
C3—C4—H4A	120.0	H16A—C16—H16B	109.5
C5—C4—H4A	120.0	C15—C16—H16C	109.5
C4—C5—C6	121.18 (11)	H16A—C16—H16C	109.5
C4—C5—H5A	119.4	H16B—C16—H16C	109.5
C6—C5—H5A	119.4	O3—C17—H17A	109.5
C5—C6—C1	117.97 (11)	O3—C17—H17B	109.5
C5—C6—C7	124.27 (11)	H17A—C17—H17B	109.5
C1—C6—C7	117.67 (11)	O3—C17—H17C	109.5
N2—C7—N1	112.04 (10)	H17A—C17—H17C	109.5
N2—C7—C6	121.88 (11)	H17B—C17—H17C	109.5
N1—C7—C6	126.07 (11)	N1—C18—C19	110.49 (10)
N1—C8—C9	132.13 (11)	N1—C18—H18A	109.6
N1—C8—C13	105.44 (10)	C19—C18—H18A	109.6
C9—C8—C13	122.43 (11)	N1—C18—H18B	109.6
C10—C9—C8	116.70 (11)	C19—C18—H18B	109.6
C10—C9—H9A	121.7	H18A—C18—H18B	108.1
C8—C9—H9A	121.7	O4—C19—C18	111.58 (10)
C9—C10—C11	121.52 (12)	O4—C19—H19A	109.3
C9—C10—H10A	119.2	C18—C19—H19A	109.3
C11—C10—H10A	119.2	O4—C19—H19B	109.3
C12—C11—C10	121.19 (11)	C18—C19—H19B	109.3
C12—C11—C14	116.97 (11)	H19A—C19—H19B	108.0
C10—C11—C14	121.83 (11)	H5B—O5—H5C	107.1 (19)
			
C6—C1—C2—C3	0.6 (2)	N1—C8—C9—C10	179.73 (12)
C17—O3—C3—C4	9.95 (18)	C13—C8—C9—C10	−1.15 (17)
C17—O3—C3—C2	−170.04 (11)	C8—C9—C10—C11	0.95 (17)
C1—C2—C3—O3	178.34 (12)	C9—C10—C11—C12	0.34 (18)
C1—C2—C3—C4	−1.65 (19)	C9—C10—C11—C14	179.15 (11)
O3—C3—C4—C5	−179.16 (11)	C10—C11—C12—C13	−1.45 (17)
C2—C3—C4—C5	0.83 (19)	C14—C11—C12—C13	179.70 (10)
C3—C4—C5—C6	1.05 (18)	C7—N2—C13—C12	−179.60 (12)
C4—C5—C6—C1	−2.05 (18)	C7—N2—C13—C8	0.40 (13)
C4—C5—C6—C7	−178.52 (11)	C11—C12—C13—N2	−178.75 (11)
C2—C1—C6—C5	1.22 (18)	C11—C12—C13—C8	1.25 (16)
C2—C1—C6—C7	177.92 (11)	N1—C8—C13—N2	−0.62 (13)
C13—N2—C7—N1	−0.02 (13)	C9—C8—C13—N2	−179.95 (10)
C13—N2—C7—C6	178.74 (10)	N1—C8—C13—C12	179.38 (10)
C8—N1—C7—N2	−0.37 (13)	C9—C8—C13—C12	0.05 (17)
C18—N1—C7—N2	176.36 (11)	C15—O1—C14—O2	2.88 (18)
C8—N1—C7—C6	−179.06 (11)	C15—O1—C14—C11	−177.56 (10)
C18—N1—C7—C6	−2.33 (19)	C12—C11—C14—O2	12.75 (18)
C5—C6—C7—N2	147.34 (12)	C10—C11—C14—O2	−166.10 (12)
C1—C6—C7—N2	−29.13 (16)	C12—C11—C14—O1	−166.81 (10)
C5—C6—C7—N1	−34.09 (18)	C10—C11—C14—O1	14.34 (16)
C1—C6—C7—N1	149.44 (12)	C14—O1—C15—C16	−171.89 (13)
C7—N1—C8—C9	179.82 (12)	C7—N1—C18—C19	103.77 (13)
C18—N1—C8—C9	2.86 (19)	C8—N1—C18—C19	−79.98 (13)
C7—N1—C8—C13	0.59 (12)	N1—C18—C19—O4	−172.67 (10)
C18—N1—C8—C13	−176.38 (10)		

### Hydrogen-bond geometry (Å, °)

**Table:**

*Cg*2 is the centroid of the C1–C6 ring.

**Table:**

*D*—H···*A*	*D*—H	H···*A*	*D*···*A*	*D*—H···*A*
O5—H5B···O4^i^	0.86 (2)	1.98 (2)	2.8165 (15)	165 (2)
O5—H5C···N2^ii^	0.85 (1)	1.95 (1)	2.8011 (15)	175 (2)
C15—H15A···Cg2^iii^	0.97	2.95	3.7247 (17)	138

Symmetry codes: (i) −*x*+1, *y*+1/2, −*z*−1/2; (ii) *x*, −*y*−1/2, *z*−1/2; (iii) −*x*+1, −*y*, −*z*.
